# Abundant Synthesis of Netrin-1 in Satellite Cell-Derived Myoblasts Isolated from EDL Rather Than Soleus Muscle Regulates Fast-Type Myotube Formation

**DOI:** 10.3390/ijms22094499

**Published:** 2021-04-26

**Authors:** Takahiro Suzuki, Aika Mori, Takahiro Maeno, Rio Arimatsu, Emi Ichimura, Yuriko Nishi, Kouga Hisaeda, Yuki Yamaya, Ken Kobayashi, Mako Nakamura, Ryuichi Tatsumi, Koichi Ojima, Takanori Nishimura

**Affiliations:** 1 Laboratory of Muscle and Meat Science, Department of Animal and Marine Bioresource Sciences, Faculty of Agriculture, Graduate School of Agriculture, Kyushu University, Motooka 744, Nishi-ku, Fukuoka 819-0395, Japan; takatakamaeno@agr.kyushu-u.ac.jp (T.M.); mako_n@agr.kyushu-u.ac.jp (M.N.); rtatsumi@agr.kyushu-u.ac.jp (R.T.); 2 Laboratory of Cell and Tissue Biology, Research Faculty of Agriculture, Graduate School of Agriculture, Hokkaido University, Kita 9, Nishi 9, Kita-ku, Sapporo 060-8589, Japan; baumkuchen125@gmail.com (A.M.); rioari@anim.agr.hokudai.ac.jp (R.A.); e.ichi@anim.agr.hokudai.ac.jp (E.I.); y.nishi.2464@gmail.com (Y.N.); kouga225.karate@gmail.com (K.H.); ymy@anim.agr.hokudai.ac.jp (Y.Y.); kkobaya@anim.agr.hokudai.ac.jp (K.K.); nishi@anim.agr.hokudai.ac.jp (T.N.); 3 Muscle Biology Research Unit, Division of Animal Products Research, Institute of Livestock and Grassland Science, NARO, 2 Ikenodai, Tsukuba 305-0901, Japan; koojima@affrc.go.jp

**Keywords:** satellite cells, netrin-1, myotube, myofiber type, fast-twitch, myosin heavy chain

## Abstract

Resident myogenic stem cells (satellite cells) are attracting attention for their novel roles in myofiber type regulation. In the myogenic differentiation phase, satellite cells from soleus muscle (slow fiber-abundant) synthesize and secrete higher levels of semaphorin 3A (Sema3A, a multifunctional modulator) than those derived from extensor digitorum longus (EDL; fast fiber-abundant), suggesting the role of Sema3A in forming slow-twitch myofibers. However, the regulatory mechanisms underlying fast-twitch myotube commitment remain unclear. Herein, we focused on netrin family members (netrin-1, -3, and -4) that compete with Sema3A in neurogenesis and osteogenesis. We examined whether netrins affect fast-twitch myotube generation by evaluating their expression in primary satellite cell cultures. Initially, netrins are upregulated during myogenic differentiation. Next, we compared the expression levels of netrins and their cell membrane receptors between soleus- and EDL-derived satellite cells; only netrin-1 showed higher expression in EDL-derived satellite cells than in soleus-derived satellite cells. We also performed netrin-1 knockdown experiments and additional experiments with recombinant netrin-1 in differentiated satellite cell-derived myoblasts. Netrin-1 knockdown in myoblasts substantially reduced fast-type myosin heavy chain (MyHC) expression; exogenous netrin-1 upregulated fast-type MyHC in satellite cells. Thus, netrin-1 synthesized in EDL-derived satellite cells may promote myofiber type commitment of fast muscles.

## 1. Introduction

Mammalian skeletal muscle can be classified into different myofiber types based on their contractility (fast- and slow-twitch), metabolism (glycolytic and oxidative), fatigue resistance (low and high), and morphological features (e.g., colors, thickness, etc.). The specific myofiber type and its maintenance system are generally affected by the frequency of motor nerve impulse [1]. Motor neurons are categorized as slow and fast type as well as myofiber type [2]. The intracellular mechanisms underlying the myofiber type regulation has been revealed; a transcriptional circuitry element centered around the nuclear receptor peroxisome proliferator-activated receptor (PPAR)-delta and the co-activator PGC1alpha complex, the signaling of calcineurin-dependent nuclear factor of activated T-cells (NFAT) transcription factors, and the coordinated regulations of gene programs by micro RNAs (miRNAs) mainly stimulate myofiber commitment [1,3]. These motor-neural mechanisms principally demonstrate the myofiber type determination systems during embryonic development and postnatal growth period. Nonetheless, the regulation mechanisms of the newly formed myofiber types during the regeneration and hypertrophy of matured skeletal muscles are still unclear.

Satellite cells are resident myogenic stem cells positioned beneath the basal lamina of mature myofibers. These cells differentiate to form nascent myofibers (myotubes) or fuse to injured myofibers for skeletal muscle regeneration and hypertrophy [4,5]. According to previous studies, the functional differences in satellite cells are associated with each myofiber type; cell number per single myofiber, self-renewal potential, and proliferation and differentiation capabilities may vary depending on their residence [6,7,8,9]. Moreover, satellite cell-derived myoblasts isolated from fast- or slow-twitch muscles form myotubes, which show high expression of specific myofiber type markers based on their original muscles [9,10]. These reports suggest that satellite cells may autonomously regulate myofiber type before the onset of nerve control during the regeneration of adult skeletal muscles. We have previously reported that satellite cells synthesize and secrete the multi-potent modulator semaphorin 3A (Sema3A), a class 3 vertebrate-secreted semaphorin originally identified as a neural chemorepellent, during early myogenic differentiation in response to in vivo injury (by crush or cardiotoxin), as well as in primary cultures in vitro [11,12,13,14]. The comparative analysis in adult rat satellite cells revealed the higher expression level of Sema3A in soleus muscle (abundant in slow-twitch myofiber) than in extensor digitorum longus (EDL) muscle, a fast-twitch muscle [15]. We have also found that the interaction between satellite cell (myoblast)-derived Sema3A and its cell-membrane receptor complex (neuropilin2-plexinA3) impacts the slow-twitch myofiber commitment in myogenic differentiation models in vivo and in vitro [16]. These reports suggest that satellite cells play key roles in autonomous myofiber type commitment systems through the synthesis of multi-potent modulators during skeletal muscle regeneration. Nevertheless, there is no evidence on whether the fast-twitch myofiber commitment model was also regulated by satellite cells, as observed for slow-twitch fibers.

To investigate the above question, we chose to profile new candidates from other multi-potent modulators, originally identified as axon-guidance cue ligands (ephrins, netrins, and slits) synthesized in satellite cells (myoblasts), including their receptors (Ephs, DCC, neogenin, Unc5s, and Robos) [17,18]. Here, we focus on the netrin family of proteins and their receptors as modulators of fast-twitch myofiber type, as they compete with Sema3A during neurogenesis and osteogenesis. In mammals, netrins are categorized as three secreted proteins (netrin-1, -3, and -4) and two membrane-tethered glycophosphatidylinositol (GPI)-linked proteins (netrin-G1 and -G2). The receptors for secreted netrins are deleted in colorectal cancer (DCC), the DCC paralogue (neogenin), and the UNC-5 homologues (UNC5A-D) [19]. Although the physiological functions of netrin proteins in the nervous system vary depending on the ligand-receptor interaction, secreted netrins were first identified as “chemoattractants” of axon guidance molecules. Sema3A, on the other hand, was termed as a “chemorepellent” [20]. In the osteo system, the interaction between netrin-1, one of the secreted netrins, and its receptor Unc5B promoted osteoclast differentiation [21], whereas that between Sema3A and its receptor neuropilin-1 increased osteoblastic bone formation and suppressed osteoclastic bone resorption to mediate an osteoprotective effect [22]. Furthermore, netrin-3 and neogenin promoted myoblast differentiation and myotube formation [17]. Neogenin was shown to interact with cell membrane proteins, cell adhesion molecule-related/downregulated by oncogenes (CDO), and bioregional cell adhesion molecule-related/downregulated by oncogene-binding protein (BOC), to promote myogenic differentiation signaling [23,24]. These reports encouraged us to hypothesize that the interaction of secreted-type netrins with their receptors on satellite cells (myoblasts) is the key factor for fast-twitch myofiber commitment, as opposed to that with Sema3A as a slow-twitch myofiber modulator.

To test our hypothesis, we first assessed the expression pattern of netrin-1, -3, and -4 in satellite cell primary cultures and found that netrin-1 and -4 were substantially upregulated during myogenic differentiation. Comparative studies of the expression levels of netrins and their receptors in soleus- and EDL-derived satellite cells demonstrated that only netrin-1 was highly expressed in EDL-derived satellite cells of our primary cultures. We performed specific knockdown experiments in satellite cell-derived myoblast cultures to identify whether netrin-1 synthesized by satellite cells regulates myofiber type, as observed with Sema3A. Netrin-1 knockdown clearly suppressed the expression levels of fast-twitch myofiber markers through the inhibition of myotube formation but had little effect on slow-twitch myotube formation. We also assessed whether netrin-1 potentially regulates myofiber type by performing an additional experiment with recombinant netrin-1 in the differentiated satellite cell-myoblast cultures. Exogenous netrin-1 not only upregulated the expression levels of fast-twitch myofiber markers, but also strongly downregulated the slow-twitch markers. These results suggest that satellite cells isolated from EDL muscle autonomously promote fast-twitch myotube formation by overexpressing netrin-1 than those isolated from soleus muscle.

## 2. Results

### 2.1. Netrin-1 and -4 Expression Levels Are Upregulated in Satellite Cells during Myogenic Differentiation

**Figure 1:**
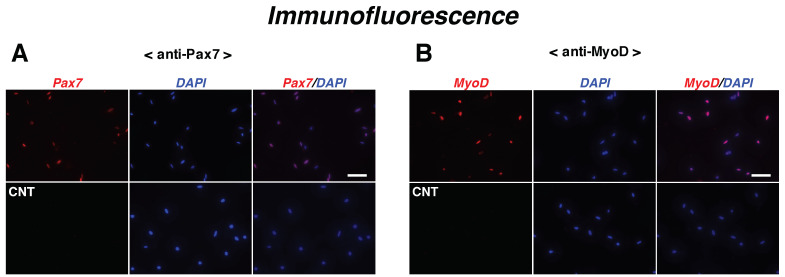
Myogenic capability of satellite cells. (**A**,**B**) Satellite cell primary cultures isolated from the back, buttock, and upper hind limb were maintained for 24 h in F-10–20% FBS medium and evaluated for the ratio of cells expressing each myogenic protein. Immunofluorescence microscopy for Pax7 (panel **A**; red) or MyoD (panel **B**; red) and DAPI (blue) showing satellite cells and nuclei. **CNT**, control cells stained without primary antibodies. Scale bar in panels (**A**,**B**), 50 μm.

To examine the synthesis pattern of netrins (netrin-1, -3, and -4) in satellite cells during myogenic differentiation, we prepared primary cultures of satellite cells derived from 2-month-old male mice. To investigate their myogenic capability, the cells expressing myogenic cell markers (paired box protein 7 [Pax7] and basic helix-loop helix myogenic transcription factor MyoD) were first detected by immunocytochemistry (Figure 1A,B). The percentage of cells expressing Pax7 and MyoD were 90.3% ± 1.57% and 95.0% ± 0.73% (means ± standard error of means [SEM] for three independent cultures), respectively, suggesting that this primary culture system offered favorable conditions to examine the myogenic capability of satellite cells.

**Figure 2:**
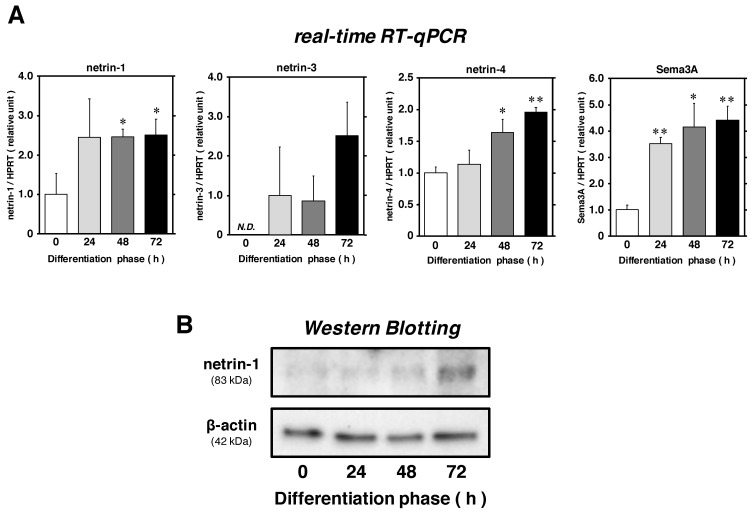
The expression levels of netrins were upregulated in satellite cells during myogenic differentiation. Satellite cell primary cultures, prepared as shown in Figure 1, were maintained in differentiation medium (DM) after the induction of proliferation. (panel **A**) The mRNA expression of netrin-1, -3, and -4 and Sema3A was evaluated by real-time RT-qPCR normalized to the level of HPRT at all time points indicated. The bars depict the mean ± SEM of three independent cultures by relative units, as compared to differentiation at 0 h. As netrin-3 expression could not be detected at 0 h, relative units were compared to the 24 h time point. Significant differences from the mean of 0 h (the left-most open bar) at *p* < 0.05 and *p* < 0.01 are indicated by single and double asterisks, respectively. (panel **B**) The protein expression of netrin-1 was detected with ECL-Western Blotting standardized to β-actin expression level.

Proliferating satellite cells (approximately 50–60% confluency) were incubated in Dulbecco’s modified Eagle’s medium (DMEM) supplemented with 5% horse serum (HS) as differentiation medium (DM) until 72 h. The cell lysates were analyzed for the expression of netrins and Sema3A with real-time reverse-transcription quantitative polymerase chain reaction (real-time RT-qPCR) and Western Blotting at each time point (Figure 2A,B). Consistent with the expression pattern of Sema3A, netrin-1 mRNA expression level relative to that of hypoxanthine guanine phosphoribosyl transferase (HPRT) was substantially upregulated and reached a maximum (approximately 2.5 times higher than that in differentiation phase at 0 h). The protein expression of netrin-1 was also clearly upregulated at 72 h. A gradual upregulation in the mRNA expression of netrin-4 was also seen until 72 h (approximately 2.0-fold). While netrin-3 mRNA expression showed no significant changes, we believe that it may slowly be upregulated, as observed in a previous report using C2C12 mouse myoblasts during the induction of myogenic differentiation [17]. These data provide an evidence that satellite cells can synthesize and upregulate the expression levels of netrins during myogenic differentiation in a time-dependent manner.

### 2.2. Satellite Cells Isolated from EDL Synthesize Higher Levels of Netrin-1 Than Those from Soleus

**Figure 3:**
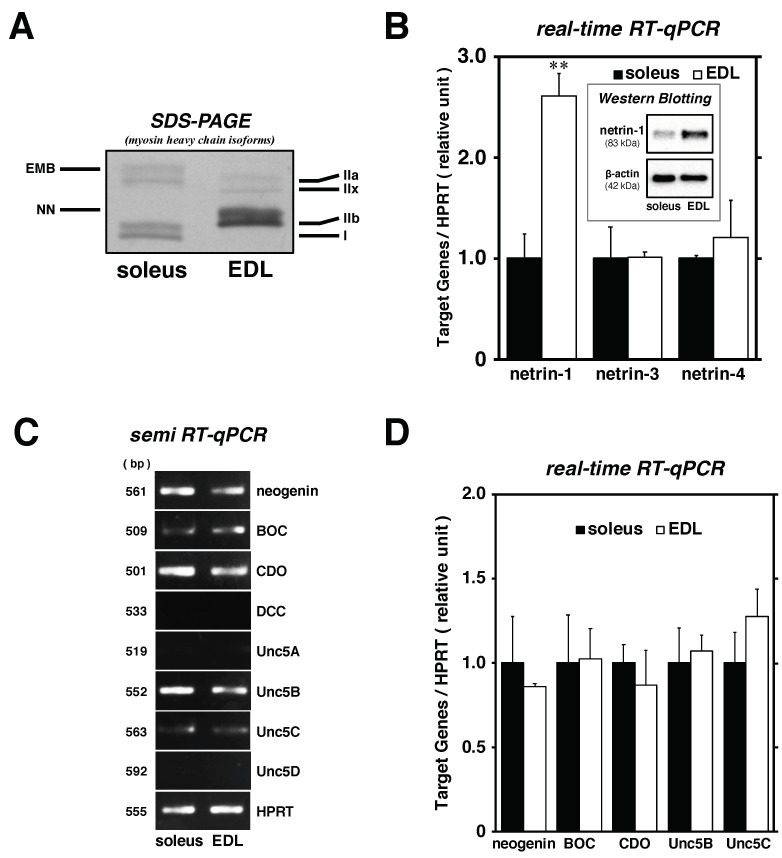
Comparative analysis of netrin expression levels in EDL and soleus muscle-derived satellite cells. (panel **A**) Statues of soleus and EDL muscle; myofiber type composition of the skeletal muscle. MyHC isoforms (myofiber type; I [slow-twitch], IIa [intermediate], and IIx and IIb [fast-twitch]) were separated using SDS polyacrylamide gel electrophoresis (SDS-PAGE) of whole muscle samples (100 ng protein) and then visualized by silver staining. (panel **B**–**D**) Proliferated satellite cells from soleus and EDL muscles were maintained in differentiation medium until 72 h. (panel **B** and **D**) The cell lysates were evaluated for the mRNA expression of netrin-1, -3, and -4, neogenin, BOC, CDO, Unc5B, and Unc5C by real-time RT-qPCR standardized to HPRT expression. The bars depict the mean ± SEM of three independent cultures by relative units. (panel **B** inset) Netrin-1 protein expression by Western Blotting standardized to β-actin expression. (panel **C**) The cell lysates were analyzed for the expression of netrin receptors (neogenin, BOC, CDO, DCC, Unc5A, Unc5B, Unc5C, and Unc5D) by routine RT-semiquantitative PCR standardized to HPRT level. Significant differences from satellite cells from soleus (black bars) at *p* < 0.01 are indicated by double asterisks. **EMB**: embryonic isoform; **NN**: neonatal isoform.

One of the main purposes of this study was to examine whether netrin proteins would serve as modulators of myofiber types in myotubes, consistent with the function of Sema3A [16]. We compared the synthesis patterns of netrins and their receptors between soleus muscle-derived and EDL-derived satellite cells. As each satellite cell was prepared from a 2-month-old male mouse skeletal muscle in this study, the myofiber types in the soleus and EDL were investigated for myosin heavy chain (MyHC) isoforms [25]. As a result, MyHC type I as slow MyHC was clearly detected in the soleus muscle but not in the EDL, while all MyHC type II isoforms (IIa, IIx, and IIb) as fast MyHC were expressed in the EDL; only type IIa which is recognized as an intermediate isoform in type II MyHCs could be detected in the soleus muscle (Figure 3A). These data provide a suitable background of the composition of soleus and EDL muscle (slow- and fast-twitch muscle, respectively) in mice. Bands of MyHC isoforms detected in the gel electrophoresis assay were categorized into six isoforms in the order of increasing mobility [26].

Each satellite cell isolated from soleus and EDL muscle was maintained for 72 h in DMEM-5% HS after proliferation and compared for the expression levels of netrins (netrin-1, -3, and -4) with real-time RT-qPCR and Western Blotting (Figure 3B). The mRNA expression of netrin-1 in EDL-derived satellite cells was clearly higher (approximately 2.6-fold) than that detected in soleus-derived satellite cells; the same pattern was confirmed in the protein expression level normalized to β-actin expression. However, no significant difference was observed in the expression levels of both netrin-3 and -4 at this myogenic differentiation stage. We performed comparative analysis of the cell membrane receptors of netrin proteins (neogenin, BOC, CDO, DCC, Unc5A, Unc5B, Unc5C, and Unc5D) between soleus- and EDL-derived satellite cells, as netrins are secretory proteins and require ligand-binding components to mediate their physiological functions during myogenesis and in various organs, including the nerve, vasculature, lung, pancreas, and mammary gland (Figure 3C,D) [17,19,23,24,27,28,29]. Agarose gel electrophoresis of semi-RT-qPCR products showed that the mRNAs encoding neogenin, BOC, CDO, Unc5B, and Unc5C were detected in both soleus- and EDL-derived satellite cells, but no significant difference was observed with real-time RT-qPCR. These data suggest that netrin-1, abundantly synthesized in the satellite cells isolated from EDL muscle, would be a potential myofiber type modulator for fast-twitch myotube commitment. 

### 2.3. Netrin-1 Specifically Affects the Expression of Fast-MyHC during Myotube Formation Process

**Figure 4:**
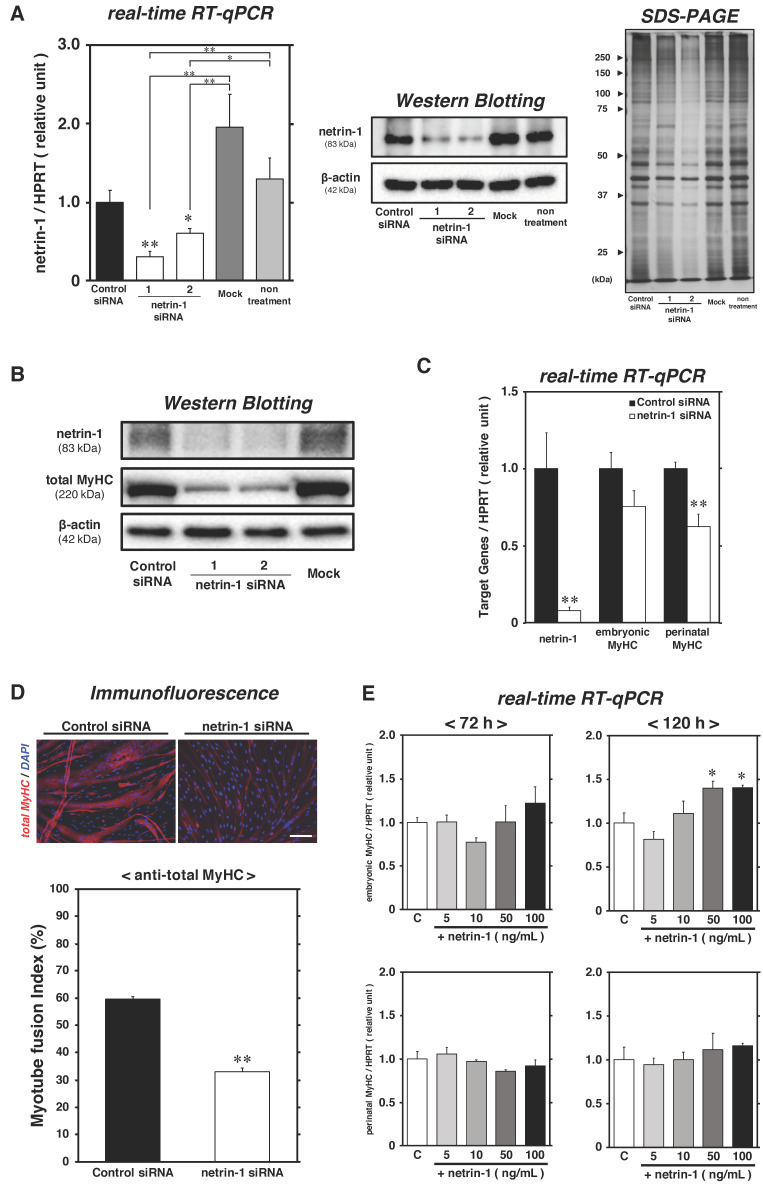
Myotube formation was regulated by netrin-1 during myogenic differentiation. The satellite cell-derived myoblasts were grown in the PM for 24 h, then the medium was replaced with DM, myoblasts were transfected with netrin-1 siRNAs and were incubated in the DM for up to 72 h or 120 h. (panel **A**–**C**) The mRNA expression levels of netrin-1 at 72 h (shown in panel **A**), netrin-1, embryonic MyHC, and perinatal MyHC at 120 h (shown in panel **C**) were evaluated by real-time RT-qPCR and normalized to the level of HPRT. The bars depict the mean ± SEM of three independent cultures by relative units, as compared to control siRNA-treated cultures. The protein expression levels of netrin-1 at 72 h (shown in panel **A**), and those of netrin-1 and total MyHC (shown in panel **B**) were also detected with Western Blotting using cell lysates; β-Actin was used for protein expression normalization. (panel **A** right-side image) Following Western Blotting analysis, equivalent amounts of whole cell lysates from each culture group at 72 h were subjected to SDS-PAGE, and subsequently visualized by silver staining. (panel **D**) Immunofluorescence microscopy for total MyHC (red) and DAPI (blue) shows myotubes and nuclei. Scale bar in panel, 100 μm. Myotube fusion index was evaluated and defined as the ratio of myonuclei on myotubes containing more than two nuclei. The results are expressed as the mean ± SEM of three independent cultures. Significant differences from the means of control siRNA cultures (black bar) at *p* < 0.05 or *p* < 0.01 are indicated by single or double asterisks. (panel **E**) Satellite cell primary cultures, prepared as shown in Figure 2, were differentiated in DM for 48 h after the induction of proliferation. Then, satellite cells were maintained for 24 h or 72 h (beginning at 48 h post-differentiation in DM) in media containing recombinant netrin-1 (0, 5, 10, 50, and 100 ng/mL) and analyzed by real-time RT-qPCR standardized HPRT, for embryonic MyHC and perinatal MyHC mRNAs expression at 72 h or 120 h post-differentiation, respectively. The bars depict the mean ± SEM of three independent cultures by relative units, as compared to control culture. Significant differences from the control culture mean (the left-most open bar) at *p* < 0.05 is indicated by a single asterisk. **C**: control culture.

To investigate whether netrin-1 synthesized in satellite cells modulates the myofiber type of myotubes, we performed the netrin-1 knockdown experiment with specific small-interfering RNA (siRNA) transfection. The satellite cell-derived myoblasts established from C57BL/6 mice were proliferated for 24 h [16,30], and then incubated in DM with 40 nM control or netrin-1 siRNAs for 72 h or 120 h. After transfection, netrin-1 mRNA was considerably suppressed following treatment with both netrin-1 specific siRNA No. 1 (stealth_708; approximately 70% decrease at 72 h, approximately 93% decrease at 120 h) and No. 2 (stealth_1463; approximately 40% decrease at 72 h), as compared to that observed after control siRNA culture treatment; we also observed a remarkable downregulation of netrin-1 mRNA in knockdown cultures, compared to mock and non-treated cultures (Figure 4A). Netrin-1 protein expression levels had also clearly decreased at 72 h, and almost diminished at 120 h after knockdown treatment with both netrin-1 specific siRNAs (Figure 4A,B). The knockdown efficiency was weaker in netrin-1 siRNA No. 2 than in No. 1, and a small reduction in total protein was detected by SDS-PAGE in whole cell lysates collected from the No. 2 culture (Figure 4A). Based on these data, netrin-1 siRNA No. 1 was selected for subsequent real-time RT-qPCR and immunofluorescence experiments. First, we assessed the expression levels of MyHCs as myotube markers and evaluated the myotube fusion index, given that netrins play key roles in myotube formation and myogenic differentiation [17,31]. Along with netrin-1 expression, the protein expression level of total MyHC recognizing all isoforms was clearly downregulated (Figure 4B). While the suppression level of embryonic MyHC expression was not clearly detected (approximately 25% decrease), a significant downregulation of perinatal MyHC mRNA expression was also seen in netrin-1 knockdown cultures (approximately 40% decrease) (Figure 4C). Moreover, the myotube fusion index substantially decreased in netrin-1 knockdown cultures (32.9% ± 1.39%) than in control siRNA-treated cultures (59.6% ± 0.79%) (Figure 4D). 

Next, we investigated the positive effects of netrin-1 ligand on myogenic differentiation in primary satellite cell cultures. As shown in Figure 2, proliferating satellite cell primary cultures were maintained in DM until 48 h; cultures were transferred to DMs each containing recombinant netrin-1 proteins at 5, 10, 50, or 100 ng/mL concentrations from 48 to 72 or 120 h, and the expression levels of embryonic and perinatal MyHC mRNAs were evaluated by real-time RT-qPCR. Although no effects of netrin-1 ligands were observed at 72 h post-differentiation, significant upregulation of embryonic MyHC by increasing concentrations of netrin-1 was observed and reached a maximum between 50 and 100 ng/mL (approximately 1.4-fold higher than the mean in control cultures without recombinant netrin-1) at 120 h (Figure 4E). We expected that the expression levels of perinatal MyHC mRNA would also be upregulated by netrin-1, as predicted from the results of the knockdown experiment above, but no significant differences were observed in each netrin-1 addition culture.

These results suggest that netrin-1 synthesized in satellite cells during myogenic differentiation promotes myotube formation.

**Figure 5:**
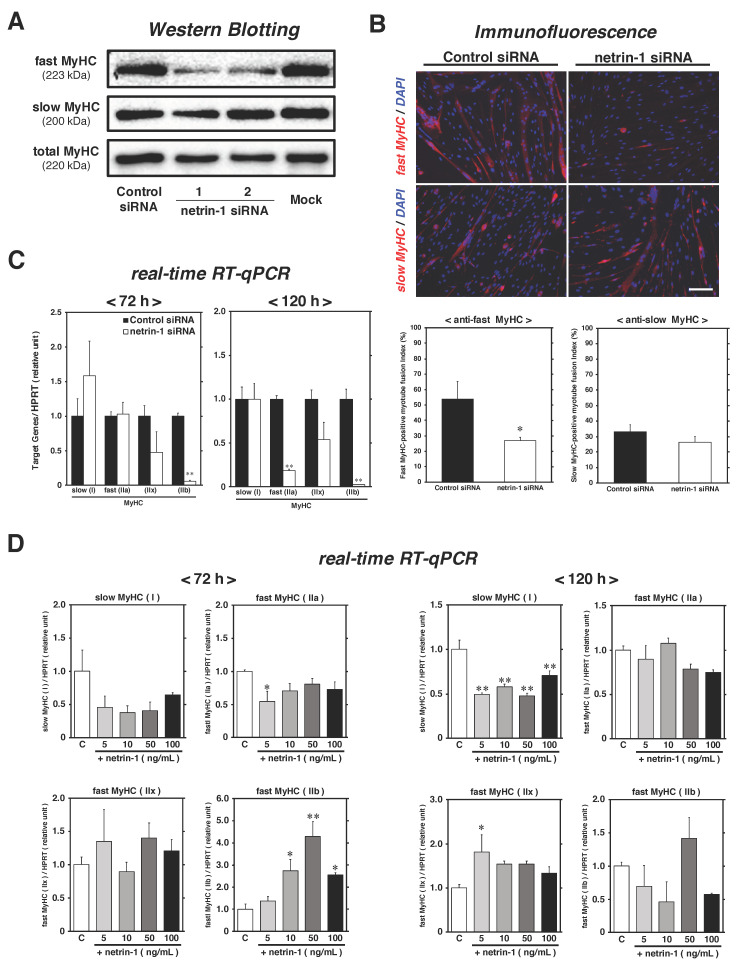
Significant reduction in the expression of fast-MyHC was observed following netrin-1 expression knockdown, and in the slow-MyHC expression level was found following netrin-1 addition to satellite cells. As described in Figure 4, proliferating satellite cell-derived myoblasts were maintained in DM with netrin-1 siRNA until 72 h or 120 h. (panel **A**) The protein expression of fast MyHC (contained type IIa, IIx, and IIb) and slow MyHC (type I) was detected with Western Blotting using cell lysates after normalization to the expression level of total MyHC. (panel **B**) Immunofluorescence microscopy for fast/slow MyHC (red) and DAPI (blue) shows fast-/slow-twitch myotubes and nuclei. Scale bar in panel, 100 μm. Myotube fusion index of each myofiber type was evaluated and defined as the ratio of myonuclei on myotubes containing more than two nuclei. The results are expressed as the mean ± SEM of three independent cultures. (panel **C**) The cell lysates were analyzed for the mRNA expression of MyHC type I, IIa, IIx, and IIb by real-time RT-qPCR standardized to HPRT. The bars depict the mean ± SEM of three independent cultures by relative units, as compared to control siRNA-treated cultures. Significant differences from control siRNA cultures (black bar) at *p* < 0.05 or *p* < 0.01 are indicated by single or double asterisks. (panel **D**) As well as in Figure 4E, satellite cell primary cultures in DM were respectively added recombinant netrin-1 (0, 5, 10, 50, and 100 ng/mL) at 48 h post-differentiation and maintained until 72 h or 120 h. The mRNA expression levels of MyHC type I, IIa, IIx, and IIb isoforms were evaluated by real-time RT-qPCR and were normalized to the level of HPRT at all cultures indicated. The bars depict the mean ± SEM of three independent cultures by relative units, as compared to control culture. Significant differences from the control culture (the left-most open bar) at *p* < 0.05 and *p* < 0.01 are indicated by single and double asterisks, respectively. **C**: control culture.

To confirm the composition of myofiber types in myotubes following netrin-1 expression knockdown treatment, the expression patterns of slow/fast MyHC as myofiber type markers were evaluated using Western Blotting following normalization to total MyHC expression at 120 h of differentiation (Figure 5A). No differences in slow MyHC expression levels were observed among all cultures, whereas fast MyHC was specifically downregulated in netrin-1 knockdown cultures. At the same time point, evaluation of myotube fusion index for each myofiber type revealed that the percentage of fast MyHC-positive myotubes was substantially lower in netrin-1 knockdown cultures (26.7% ± 2.73%) than in control siRNA-treated cultures (53.7% ± 11.47%), but the ratio of slow MyHC was similar between the control (33.1% ± 4.40%) and netrin-1 siRNA treatment groups (26.2% ± 3.72%) (Figure 5B). The results of mRNA expression patterns of myofiber type specific MyHC isoforms, as detected by real-time RT-qPCR standardized HPRT, showed that type IIb was stably suppressed in netrin-1 knockdown cultures at both differentiation time points (72 h and 120 h) (Figure 5C). The analysis data of type IIa mRNA expression level was also considerably lower in knockdown cultures than in control cultures at 120 h. These data suggest that the ability of myoblasts in netrin-1 knockdown cultures to form fast-twitch myotubes was drastically inhibited.

Moreover, we also assessed whether the addition of exogenous netrin-1 to primary satellite cell cultures may regulate myofiber types (Figure 5D). As shown in Figure 4E, we prepared primary satellite cell cultures and induced myogenic differentiation in DM containing recombinant netrin-1 proteins at different concentration (0, 5, 10, 50, and 100 ng/mL). The muscles we chose for satellite cell isolation in this experiment were a mix of the back, buttock, and upper hind limb muscle groups, each of which, contained fast-twitch myofibers, mainly categorized as type IIb fibers (data not shown). The mRNA expression levels of MyHC type I were stably inhibited between 72 h and 120 h post-differentiation. Especially at 120 h, a significant downregulation of type I was observed in each netrin-1 addition culture, compared to the control culture (approximately 45% less on average). Following type I, we investigated the mRNA expression patterns of MyHC type II isoforms, guessing contrary upregulation. At 72 h post-differentiation, the mRNA expression level of type IIa was downregulated in 5 ng/mL netrin-1 containing culture (approximately 45% less), while type IIb expression levels in 10, 50, and 100 ng/mL cultures were clearly higher than those in the control culture (approximately 2.5–4.5-fold). Although we could not observe the drastic upregulation of type II isoform expression by exogenous netrin-1 at 120 h, type IIx was substantially higher in the 5 ng/mL netrin-1 containing culture than in the control (approximately 1.8-fold).

Taken together, these results show that netrin-1 may be responsible for fast-twitch myotube commitment in satellite cell cultures prepared from EDL muscle, as compared to those prepared from soleus.

## 3. Discussion

Previously published studies have shown that the expression of cellular signaling factors such as focal adhesion kinase (FAK) and extracellular signal-regulated kinase (ERK) is enhanced by the interaction between netrins (mainly netrin-3) and their receptors (neogenin/CDO) in myoblasts and is important for myogenic differentiation and myotube formation [17,28,29]. Here, we first confirmed the upregulation in the expression of netrins (especially netrin-1) in satellite cells during myogenic differentiation along with Sema3A expression (Figure 2). The knockdown of netrin-1 following siRNA treatment of satellite cell-derived myoblasts resulted in the downregulation of total MyHC protein and perinatal MyHC mRNA expression, and inhibited myotube formation (Figure 4B–D). We also observed that satellite cell primary cultures maintained with recombinant netrin-1 protein highly expressed embryonic MyHC mRNA, compared to the control culture (Figure 4E). These results are consistent with those reported in previous studies and suggest that netrin-3, as well as netrin-1 synthesized in satellite cells (myoblasts), perform key functions to form myotubes in an autonomous manner [17,31]. Our future studies will be directed to identify the specific receptors of netrin-1 in satellite cells and determine the cellular signaling cascades.

To compare the expression levels of netrins and their receptors in slow- and fast-twitch muscle-derived satellite cells during differentiation, we separately isolated satellite cells from soleus and EDL muscles and performed comparative studies. Only netrin-1 mRNA and protein levels were higher in satellite cells from EDL muscle than in those isolated from soleus (Figure 3B). Together, with the results highlighting the role of netrin-1 as an accelerator for myotube formation (Figure 4), our data support the previous finding that the fast-type muscle-derived myoblasts have a higher capability to form myotubes than slow-type muscle-derived cells [9]. We predict that netrin-1 synthesis patterns may affect fast-twitch myotube commitment because our previous study showed that the expression and secretion level of Sema3A as a major slow-twitch myofiber determination factor in satellite cells was clearly opposite to that of netrin-1 [15]. Although the mRNA expression of other netrins and secreted netrin receptors (neogenin, BOC, CDO, Unc5B, and Unc5C) was detected, we failed to confirm any difference in their expression levels between soleus- and EDL-derived satellite cells (Figure 3B–D). As the comparative study was performed only at one time point (differentiation for 72 h), we comprehensively profiled the expression patterns of all netrin proteins and their receptors in each satellite cell during different differentiation phases. Our preliminary experiments revealed that the protein expression levels of netrin-1 and -4 were consistently higher in EDL-derived satellite cells than in soleus-derived cells during late differentiation stages (data not shown). In the subsequent results of knockdown experiments and recombinant protein addition treatment, we directly confirmed netrin-1 as a novel myofiber type modulator produced in satellite cells. The protein expression levels of fast MyHC in myotubes and the fusion index of fast MyHC-positive myotubes were specifically lower in netrin-1 siRNA-transfected cultures than in control siRNA-treated and mock cultures (Figure 5A,B). In contrast, no influence was observed on slow-twitch myotube formation. The mRNA expression patterns of MyHC isoforms suggest that MyHC type IIb, the most fast-twitch isoform, was the specific target of the synthesized netrin-1-dependent modulation system during myogenic differentiation (Figure 5C). Following the recombinant netrin-1 addition experiment in differentiated satellite cell primary cultures derived from fast-type muscle groups, exogenous netrin-1 upregulated MyHC type IIb and regulated other isoforms to promote fast-type myotube formation (Figure 5D). In particular, the mRNA expression level of MyHC type I, the most slow-twitch isoform, was clearly inhibited in netrin-1 addition cultures. Taken together, the results of this study strongly suggest that netrin-1, abundantly synthesized in EDL-derived satellite cells, plays an essential role in the formation of fast-twitch myotubes. Further studies are warranted to identify the detailed cellular pathways underlying this novel regulatory mechanism. As netrin-1 requires calcineurin/NFAT-mediated transcription in axonal outgrowth and neogenin enhances NFAT-dependent transcription of C2C12 myoblasts, we will focus on the interactions of the netrin-1/fast MyHC signaling axis with the crucial intracellular mediators for myofiber type commitment, such as NFATs coordinating MyHC genes [1,17,32].

Numerous studies focusing on cell dynamics, such as quiescence, activation, proliferation, and differentiation, have shown that satellite cells exhibit heterogeneities based on the expression patterns of key transcription factors and cellular membrane proteins and determine the cell fate/linage [33,34]. Nonetheless, satellite cells have been thought to contribute to successful regeneration and hypertrophy by fusing with each other or the pre-existing myofibers without selecting any myofiber type in matured skeletal muscles. However, several studies have directly focused on characterization based on myofiber types. Satellite cells isolated from slow-twitch muscles (soleus) or fast-twitch muscles (EDL and tibialis anterior muscle) have unique potentials and form myotubes with specific myofiber type [6,9,35]. Our recent study revealed the high expression of Sema3A in soleus muscle derived-satellite cells as an essential modulator for the slow-twitch myofiber type formation during skeletal muscle regeneration via a novel signaling axis, Sema3A-neuropilin2/plexinA3-myogenin/myocyte enhancer factor 2D (MEF2D)-slow MyHC [15,16]. Here we assumed that netrin-1 modulates fast MyHC expression via MyoD, a member of the myogenic transcription factor, as well as myogenin and MEF2D because previous reports have shown that MyoD is highly expressed in EDL muscle tissue and regulates fast-twitch muscle differentiation during development [36,37,38].

The myogenic significance of multifunctional modulators, including netrins and semaphorins, originally found as axon guidance molecules has been gradually revealed. Ephrins and their receptor Ephs expressed in satellite cells regulate cell motility and myotube patterning, especially ephrin-A3 (expressed only in slow-twitch myofibers) and EphA8 (exclusively expressed in fast-type motor endplates). Their interactions at the neuromuscular junctions promote and maintain the slow-twitch myofiber commitment [39,40]. Robos, cell membrane receptors of the axon guidance molecule Slits, were also detected in satellite cells, although their myogenic function is still unclear [18]. Taken together, we may anticipate that satellite cells localized in each muscle may distinctively produce these multipotent modulators for autonomous myofiber type regulation.

## 4. Materials and Methods

### 4.1. Animal Care and Use

All experiments involving animals were conducted in strict accordance with the recommendations of the Guidelines for Proper Conduct of Animal Experiments published by the Science Council of Japan and ethics approvals from the Hokkaido University Institutional Review Board (approval No. 15-0173 and 16-0066) and from Kyushu University Institutional Review Board (approval No. A21-039). C57BL/6 mice were purchased from CLEA-Japan (Tokyo, Japan). All mice were bred and housed at 23 ± 2 °C and 55% ± 10% humidity on a 12 h light/dark cycle (lights on at 10 a.m.) and had free access to regular food (Labo MR Stock, Nosan Corporation, Yokohama, Japan) and water.

### 4.2. Satellite Cell Isolation and Primary Cultures

Satellite cells were isolated from skeletal muscles of the back, buttock, and upper hind limb (fast fiber-abundant) or soleus and EDL of 2-month-old mice according to previous reports with a few modifications, including the addition of hemolysis treatment [11,15,16,41,42]. Briefly, muscles were excised, and trimmed of the tendon, adipose tissue, vessels, and connective tissue. The tissue was minced with scissors and digested for 1 h at 37 °C with 1.0 mg/mL protease type XIV (Sigma-Aldrich, St. Louis, MO, USA). Cells were separated from muscle fiber fragments and tissue debris by differential centrifugation and filtration through PET mesh cell strainers (100 μm mesh size). Erythrocytes in cell suspensions were eliminated with Tris-buffered ammonium chloride treatment for 3 min at 4 °C, followed by centrifugation at 1700× *g* for 4 min. Cells were re-filtered through cell strainers (40 μm mesh size) prior to the final centrifugation step at 430× *g* for 10 min. Satellite cells were seeded into 12/24-well plates (IWAKI brand Asahi Glass, Tokyo, Japan) coated with poly-L-lysine (IWAKI) and fibronectin (Sigma-Aldrich) in Ham’s F-10 Nutrient Mixture medium (GIBCO, Grand Island, NY, USA) containing 20% fetal bovine serum (FBS; Biosera, Boussens, France), 0.5% gentamicin reagent (GIBCO), and 1% antibiotic-antimycotic solution (AA-mix; GIBCO) as growth medium until they reached approximately 50–60% confluency. Primary cultures were transferred in DMEM (GIBCO) containing 5% HS (GIBCO), 0.5% gentamicin reagent, and 1% AA-mix as the DM for 24–120 h. In Figure 4E and Figure 5D, satellite cells (myoblasts), isolated from the back, buttock, and upper hind limb muscles, were incubated in DM containing recombinant mouse netrin-1 (R&D Systems, Minneapolis, MN, USA) at 48 h post-differentiation, and maintained for 24 h or 72 h (until 72 h or 120 h post-differentiation). All cultures were maintained in a humidified atmosphere of 5% CO_2_ at 37 °C.

### 4.3. Knockdown of Netrin-1 Expression by siRNA Transfection in Myoblasts

Satellite cell-derived myoblasts (with quite high myogenic purity) were established from adult C57BL/6 mouse skeletal muscles [30]. These cells showed normal myogenic properties in knockdown experiments [16], and were seeded into 24-well plates coated with collagen type I (IWAKI) at a density 5.0 × 10^4^ cells/wells. The cells were maintained for 24 h in the GM without any antibiotic and antimycotic and then transfected with 40 nM mouse netrin-1-specific siRNAs for 72 h or 120 h using ScreenFect^TM^ siRNA Transfection Reagent (FUJIFILM Wako Pure Chemical Industries, Ltd., Osaka, Japan), according to manufacturer’s recommendation. Cultures that underwent transfection with Allstars negative control siRNA (Qiagen, Hilden, Germany) served as the “control siRNA” group. Myoblasts without siRNAs and with the transfection reagent were used as “mock” cultures. The other group as “Non treatment” cultures had myoblasts incubated as per protocol and without any transfection chemicals. Stealth RNAi siRNAs (Stealth_708 as netrin-1 siRNA No. 1 and 1463 as No. 2) designed by BLOCK-iT^TM^ RNAi Designer (Invitrogen as Thermo Fisher Scientific, Waltham, MA, USA) were used as netrin-1-specific siRNAs. The sense and antisense strands of mouse netrin-1 siRNAs are shown in Table 1.

### 4.4. Immunocytochemistry of Satellite Cells and Myotubes

Satellite cells and myotubes were fixed with 4% paraformaldehyde (PFA) in phosphate-buffered saline (PBS) for 15 min at 4 °C. Cells were treated with 0.2% polyethylene glycol mono-*p*-isooctylphenyl ether (Triton X-100; Sigma-Aldrich) for 15 min, and then blocked with 3% bovine serum albumin (BSA) in 0.1% polyethylene sorbitan monolaurate (Tween 20)-Tris-buffered saline (TBS-T) for 40 min at room temperature (RT; 25 °C) before incubation with a primary monoclonal antibody (1:25–50 dilution in the blocking solution, overnight at 4 °C) against Pax7 (clone Pax7; R&D Systems), MyoD (clone G-1; Santa Cruz Biotechnology, Santa Cruz, CA, USA), total MyHC (recognize all MyHC isoforms as pan-specific antibody, clone MF20; R&D Systems), fast MyHC (recognize all types IIb, IIx and IIa, clone MY-32; Sigma-Aldrich), and slow MyHC (clone NOQ7.5.4D; Sigma-Aldrich). The cells were then probed with a TRITC-conjugated goat anti-mouse IgG secondary antibody (1:250 dilution in the blocking solution for 1 h at RT; purchased from KPL, Gaithersburg, MD, USA). Cells were mounted with ProLong^TM^ Diamond Antifade Mountant with 4′,6-diamidino-2-phenylindole (DAPI) (Thermo Fisher Scientific) for microscopic observation using Leica DMI4000B confocal microscope system (Leica, Wetzlar, Germany). To count mononuclear cells expressing Pax7 or MyoD and analyze myotube fusion index, 15 images were randomly chosen per well. The myotube fusion index was calculated from the number of myonuclei localized in each MyHC isoform-positive myotubes containing two or more nuclei as percentage of total nuclei visualized with DAPI. 

### 4.5. SDS-PAGE for MyHC Isoform Composition

Soleus and EDL muscles of adult mice were analyzed for myofiber type composition following separation on 8% PAGE gels and silver staining for MyHC isoforms (types I, IIa, IIx, and IIb) [25].

### 4.6. Reverse Transcription-Polymerase Chain Reaction (RT-PCR)

Total RNA was isolated from satellite cell primary cultures or myoblasts using ISOGEN II reagent (Nippon Gene, Tokyo, Japan) according to the manufacturer’s recommendation. cDNA was synthesized from total RNA using ReverTra Ace qPCR RT Kit (TOYOBO, Osaka, Japan).

Quantitative analysis of mRNA expression level of netrin-1 (NM_008744.2), netrin-3 (NM_010947.3), netrin-4 (NM_021320.3), Sema3A (NM_009152.4), neogenin (NM_001042752.1), BOC (NM_172506.2), CDO (NM_021339.2), Unc5B (NM_029770.2), Unc5C (NM_001293561.1), embryonic MyHC (NM_001099635.1), perinatal MyHC (NM_177369.3), slow MyHC I (NM_080728.2), fast MyHC IIa (NM_001039545.2), fast MyHC IIx (NM_030679.1), and fast MyHC IIb (NM_010855.2) was carried out using LightCycler 480 Probe Master mixtures (Roche Diagnostic Ltd., Mannheim, Germany). The reaction was performed on LightCycler 480 II instrument (Roche) for TaqMan Probe-detection format and standardized using HPRT (NM_013556.2.). The primer sets were designed by Roche Probe Finder (version 2.50 and 2.53 for mouse) with an intron-spanning assay (Table 2). Annealing temperature was set to 60 °C in all cases, according to the manufacturer’s protocol. Quantitative analysis was performed using LightCycler 480 software (version 1.5.0 SP4).

Semi-reverse-transcription quantitative PCR was performed using KOD FX DNA polymerase mixture (TOYOBO) on the mRNA expression patterns of neogenin, BOC, CDO, DCC (NM_007831.3), Unc5A (NM_153131.3), Unc5B, Unc5C, and Unc5D (NM_153135.3) standardized with the expression of HPRT. The intron-spanning primer sets with amplicons and annealing temperature were designed by Primer3Plus (http://primer3plus.com/cgi-bin/dev/primer3plus.cgi, accessed on 30 June 2017), as shown in Table 3. Starting at a pre-denaturation at 94 °C for 2 min, amplification of each target cDNA was performed with 33–37 cycles (10 s denaturation at 98 °C, 30 s annealing at 61–66 °C, and 60 s extension at 68 °C). PCR products were visualized with ethidium bromide after 2.0% agarose gel electrophoresis.

### 4.7. Enhanced Chemiluminescence (ECL) and Western Blotting

Whole cell lysates from satellite cell primary cultures or myoblasts were separated on 10% PAGE gels under reducing conditions and transferred onto polyvinylidene fluoride (PVDF) membranes (FUJIFILM Wako), as previously described [15,43]. The membranes were blocked with 5% nonfat skimmed dry milk in T-TBS and incubated with antigen affinity-purified primary antibodies against netrin-1 (1:500–1000 dilution; clone No. 158936, R&D Systems), total MyHC (1:1000 dilution; clone No. MF20, R&D Systems), fast MyHC (1:500 dilution; clone No. MY-32, Sigma-Aldrich), slow MyHC (1:500 dilution; clone No. NOQ7.5.4D, Sigma-Aldrich), and β-actin (1:2000 dilution, clone No.AC-15, Abcam, Cambridge, UK) diluted in Can Get Signal solution 1 (TOYOBO) containing 0.05% sodium azide overnight at 4 °C. The membranes were then treated with horseradish peroxidase (HRP)-conjugated secondary antibodies against rat IgG (1:5000 dilution, Jackson ImmunoResearch Laboratories Inc., West Grove, PA, USA) and mouse IgG (1:5000 dilution, Jackson ImmunoResearch Laboratories Inc., West Grove, PA, USA) diluted in Can Get Signal solution 2 (TOYOBO) for 1 h at RT. The immunoreactive signals were visualized using ECL (or ECL Prime) Western Blotting Detection Reagent (GE Healthcare; Little Chalfont, UK) and the bands were captured using ChemiDoc XRS Plus luminescent image analyzer (Bio-Rad, Hercules, CA, USA).

### 4.8. Statistical Analysis

The Student’s *t*-test was used for the statistical analysis of experimental results using Microsoft Excel 2019. A one-way analysis of variance (ANOVA) test with post hoc Tukey–Kramer test in Figure 2A, Figure 4A,E and Figure 5D was performed using EZR (Saitama Medical Center, Jichi Medical University, Saitama, Japan), which is a graphical user interface for R (The R Foundation for Statistical Computing, Vienna, Austria) [44]. Data are represented as mean ± SEM for three independent cultures. Statistical significance was set at a *p* value of less than 0.05 or 0.01 indicated throughout by * or **.

## 5. Conclusions

During myotube formation, satellite cells (myoblasts) isolated from EDL muscle synthesized high levels of netrin-1 to promote fast-twitch myotube formation. In conjunction with our previous data, we showed that Sema3A considerably produced in soleus-derived satellite cells promoted slow-twitch myotube formation and that satellite cells have autonomous systems for the myofiber type regulation.

## Figures and Tables

**Table 1:** Stealth RNAi siRNA strands for mouse netrin-1 genes.

siRNA	Sense (5′ to 3′)	Antisense (5′ to 3′)
Stealth_708 (netrin siRNA 1)	GAGGUGACCUAUGUGAGCCUGCAAU	AUUGCAGGCUCACAUAGGUCACCUC
Stealth_1463 (netrin siRNA 2)	UAUCACCCACCGGAAGGCUUGCAAA	UUUGCAAGCCUUCCGGUGGGUGAUA

**Table 2:** Real-time RT-qPCR primer sets for mouse netrins, Sema3A, neogenin, BOC, CDO, Unc5B, Unc5C, MyHCs, and HPRT.

Primer Name	Primer Sequence (5′ To 3′) Forward	Primer Sequence (5′ to 3′) Reverse	Probe No.
netrin-1	CCCCTTGCATCAAGATTCC	GGCCTTGCAATAGGAGTCAC	71
netrin-3	CCCAGGTTTCCAGCAGAG	GGTCCAGGGACAGGAGTCTTA	2
netrin-4	CAGCACTGTGCACCATTGTA	TGACGTCGAAGTGACAGGTG	81
Sema3A	ATCAGTGGGTGCCTTACCAA	TCCGCCAAATGTTTTACTGG	72
neogenin	GGGGAATGAGACCAAAAATG	GGATGGGCACTAATCACAGG	83
BOC	GGACACCCATCCTGTACTACG	GAATGCCAGAAATGGTCCAG	82
CDO	GATTCCACAGAAGACACAGCAG	CCTCTGCTTCAGAATGACCAC	94
Unc5B	TTCCAGCTGCACACAACG	GCAGAGCAGAGAGCATCCA	40
Unc5C	GCACAGACCCCAGAATGAAT	CCCACGTAGAGAGCCACATC	68
embryonic MyHC	AGCCTCCAGCTTCAGAAGAA	GGCCCTCTCTGCCTCTATCT	17
perinatal MyHC	CCTGCAACTCCAGGTTCAAT	CTCTTTGATTTTGGCCTCCA	73
slow MyHC (I)	CAAGGTCAATACTCTGACCAAGG	CCATGCGCACCTTCTTCT	78
fast MyHC (IIa)	AGAGGGTTCTTGGCGAGAGT	AAGGCGCGGATGTTGTACT	22
fast MyHC (IIx)	TCTGCAGACGGAGTCAGGT	TTGAGTGAATGCCTGTTTGC	33
fast MyHC (IIb)	TGATGCAGGAGAAAAATGACC	ATCAGCTGGTCGCACCTTT	1
HPRT	CCTCCTCAGACCGCTTTTT	AACCTGGTTCATCATCGCTAA	95

**Table 3:** RT-qPCR primer sets for mouse netrin receptors and HPRT.

Primer Name	Primer Sequence (5′ to 3′) Forward	Primer Sequence (5′ to 3′) Reverse	Amplicon (bp)
neogenin	TGAATTGTGAAGTTAATGCAGATTTGGTCC	TGTCCATGGATTCGTGAGCATATATGTTAG	561
BOC	GCCGAAGCTAATATCCCAGAAAGACTATAT	ACAGAGATATGTTGCCACCGATTATTGATT	509
CDO	GGAAGGCTTTTCATACAAACGTGTGAAATA	CAAGTCAAGATGTAAGGCTACAAACTACCT	501
DCC	GTATCTACGGCTTGAAACCAGCTGAATATA	CACATAGTTCCTCGAAAGTACTTCCAGAAA	533
Unc5A	GAGTCGCCCTCTCATCTCTACTACTG	GTTAAAGTTGATGTTGAAGCTCTGTCCATC	519
Unc5B	AACTAAGTAGACAGCTGTATACCCACTCTT	CACAATCAACTTGTCTCTACTCGATTCTCA	552
Unc5C	CTATTCAGAGATATGTGCTGGTGTAAGTCC	GAGCTTAAGTGTCAGCTTTTTAGAAACAGG	563
Unc5D	TCAAGAAGGGAGATAGACCTCAAACTAGTT	CTTCCTTGGAGACATTTATTCACTCACTCA	592
HPRT	CCGAGGATTTGGAAAAAGTGTTTATTCCTC	CTTTTCCAGTTTCACTAATGACACAAACGT	555

## Data Availability

The data presented in this study are available in this article.

## Abbreviations

EDL	extensor digitorum longus
PPAR-delta	peroxisome proliferator-activated receptor-delta
NFAT	nuclear factor of activated T-cells
miRNA	micro RNA
Sema3A	semaphorin 3A
DCC	deleted in colorectal cancer
CDO	cell adhesion molecule-related/down-regulated by oncogenes
BOC	bioregional cell adhesion molecule-related/down-regulated by oncogenes binding protein
MyHC	myosin heavy chain
